# Evaluation of post-keratoplasty status with a portable and smartphone-attachable smart eye camera

**DOI:** 10.1371/journal.pone.0335450

**Published:** 2025-11-05

**Authors:** Yuto Yukari, Yuki Senoo, Takahiko Hayashi, Toshiki Shimizu, Yusuke Hara, Hiroki Nishimura, Shintaro Nakayama, Eisuke Shimizu, Satoru Yamagami

**Affiliations:** 1 Department of Ophthalmology, Nihon University Itabashi Hospital, Tokyo, Japan; 2 Emergency and General Medicine Department, Itabashi Chuo Medical Center, Japan; 3 OUI Inc., Tokyo, Japan; 4 Department of Ophthalmology, Keio University School of Medicine, Tokyo, Japan; 5 Yokohama Keiai Eye Clinic, Kanagawa, Japan; Keio University School of Medicine, JAPAN

## Abstract

**Purpose:**

To evaluate the efficacy of the smart eye camera (SEC), a portable smartphone-attachable camera, in assessing eyes following lamellar corneal transplantation.

**Methods:**

This retrospective study included consecutive patients who underwent lamellar corneal transplantation between December 1^st^ 2023 and August 31^st^ 2024. Postoperative evaluations were performed using the SEC and a slit-lamp microscope. All images from the two devices were evaluated by two corneal specialists in a blinded fashion to determine the amount of air or gas in the anterior chamber, and the presence of pupillary block and graft detachment. Sensitivity, specificity, and interrater agreement were calculated.

**Results:**

A total of 26 eyes from 23 patients (12 males and 11 females, mean age: 68.7 ± 15.2 years) were included in this study. Indications for lamellar keratoplasty included endothelial dysfunction (11 eyes were treated with Descemet membrane endothelial keratoplasty and 9 with Descemet stripping automated endothelial keratoplasty), as well as corneal opacity (3 eyes were treated with deep anterior lamellar keratoplasty). Regarding the amount of air or gas in the anterior chamber, the two raters had consistent judgments using the SEC (intraclass correlation coefficient = 0.817 [95% confidence interval: 0.616, 0.918]). A difference in pupillary block identification was found between the two examiners using the SEC, with a Kappa coefficient of 0.646. Neither examiner was able to identify the presence of graft detachment using the SEC.

**Conclusions:**

The smartphone-attachable SEC was useful in the evaluation of air/gas volume and pupillary block but not for detecting graft detachment.

## Introduction

Corneal transplantation is widely performed globally, particularly to treat eyes with partial-thickness disease, following the advent of endothelial keratoplasty and deep anterior lamellar keratoplasty (DALK) [1–4]. After corneal transplantation, the precise evaluation of graft attachment or detachment is essential, which can be achieved with imaging techniques, such as anterior segment optical coherence tomography (AS-OCT) [5,6]. However, following surgery, patients are required to remain in a supine position for a sufficient period to ensure proper graft attachment; therefore, bedside examinations may be beneficial for keeping the patient in the supine position without moving [7,8]. Furthermore, the images obtained may be shared with other doctors to determine the need for rebubbling or pressure reduction in cases of pupillary block.

Recently, smartphone camera-attachable devices for performing eye evaluations have been developed. The application of such smartphone cameras from OUI Inc. (Tokyo, Japan) has been widely reported as being very effective and useful when checking patients with cataracts in developing countries [9–12]. In this study, we aimed to evaluate the effectiveness of the smart eye camera (SEC) from OUI Inc. in assessing the status of patients following endothelial keratoplasty.

## Methods

### Study design

This study adhered to the tenets of the Declaration of Helsinki and followed all institutional guidelines. The Ethical Review Committee of Nihon University School of Medicine approved this retrospective observational study (RK-240611-2). This study included consecutive patients who underwent lamellar corneal transplantation between December 1^st^ 2023 and August 31^st^ 2024. We registered these patients on Oct 1^st^ 2024, and retrospectively reviewed medical records. We excluded patients who declined the use of SEC or missed the SEC evaluation. All data were fully anonymized before we accessed them. All patients provided informed consent in the form of opt-out on the web-site. Those who rejected were excluded.

### Surgery

All surgeries were performed by two experienced surgeons (TH and TS). Descemet membrane endothelial keratoplasty (DMEK), Descemet stripping automated endothelial keratoplasty (DSAEK), and DALK were performed using standard surgical procedures under local anesthesia. Air was injected into the anterior chamber (AC) for DALK and DSAEK procedures, while 20% sulfur hexafluoride gas was injected into the AC in DMEK.

### Patient examinations

**Fig 1:**
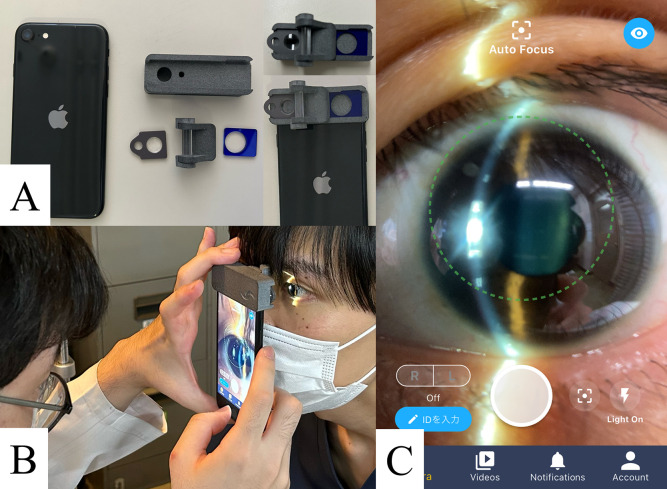
Application of the Smart Eye Camera (SEC) for Evaluating Eyes After Corneal Transplantation. (A) All parts of the SEC are shown. Following assembly, the SEC was attached to a smartphone. (B) An examiner evaluates a patient’s eye using the SEC at a close distance. (C) A picture of the patient’s eye was taken and stored on a specific digital storage platform.

All patients were examined on the day of surgery (day 0; within 3 h post-keratoplasty) using both the SEC attached to a smartphone and a standard slit-lamp microscope (Fig 1). The SEC (OUI Inc.) was attached to a smartphone to evaluate patients’ eyes and capture images, which were then stored on a digital platform. The device utilized for videorecording was the iPhone SE2 (Apple Inc.). Videos were obtained using the SEC (SLM-i07/SLM-i08SE, OUI Inc.; 13B2X10 198 030 101/13B2X10 198 030 201), and recorded at Full HD resolution (1080 × 1920 pixels) and a constant 60 Hz refresh rate [13]. The patients were examined with their eyes open. For slit-lamp examination, images were recorded using an SL130 slit lamp and a Nikon D5200 camera. All images from both devices were evaluated by two corneal specialists in a blinded fashion (YY and YH). The factors assessed included the presence of the AC, amount of air or gas in the AC, and presence of pupillary block and graft detachment. Sensitivity, specificity, and interrater agreement were calculated.

### Statistics

To determine interrater agreement, the kappa coefficient was calculated for the evaluations of graft detachment and pupillary block, while the intraclass correlation coefficient (ICC) [1,2] was calculated for the assessments of the amount of air or gas in the AC. Statistical significance was set at P < 0.05, and all analyses were performed using R version 4.2.111 [14].

## Results

### Patient demographics and surgical indications

A total of 26 eyes of 23 patients were included in the study. Patient characteristics are presented in Table 1. The indications for lamellar keratoplasty included bullous keratopathy and graft failure (treated with DMEK in 11 eyes and DSAEK in 9 eyes), as well as corneal opacity (treated with DALK in 3 eyes). In all patients, the presence of the AC and air or gas in the AC was confirmed using a slit-lamp microscope. Similar results were obtained using the SEC.

**Table 1:** Patient Characteristics.

Item (unit)	Statistics category	Calculated values (N = 23)
Age (years)	Mean (standard deviation)	68.7 (15.2)
	Median [range]	74 [25, 88]
Sex	[1] Male	12 (52.2%)
	[2] Female	11 (47.8%)
Eye	[1] Right	8 (34.8%)
	[2] Left	15 (65.2%)
Disease	[1] BK	14 (60.8%)
	[2] GF	5 (21.7%)
	[3] FECD	1 (4.3%)
	[4] Corneal opacity	3 (13%)
Surgery	[1] DMEK	11 (47.8%)
	[2] DSAEK	9 (39.1%)
	[3] DALK	3 (13%)
AC	[1] Yes	23 (100%)
Air or gas in the AC	[1] Yes	23 (100%)

Data are presented as the number (%) unless otherwise noted. BK, bullous keratopathy; GF, graft failure; FECD, Fuchs endothelial corneal dystrophy; DMEK, Descemet membrane endothelial keratoplasty; DSAEK, Descemet stripping automated endothelial keratoplasty; DALK, deep anterior lamellar keratoplasty; AC, anterior chamber.

### Evaluation of post-keratoplasty status

The SEC allowed for effective evaluation of ocular conditions following lamellar keratoplasty.

The supplemental video (S1 Video shows the eye of a patient following DMEK. In the video, the amount of air or gas in the AC, depth of the AC, and presence of graft attachment to the stroma are clearly visible.

In this study, the main factors assessed were the amount of air or gas in the AC, and presence of pupillary block and graft detachment.

### The amount of air or gas in the AC

**Fig 2:**
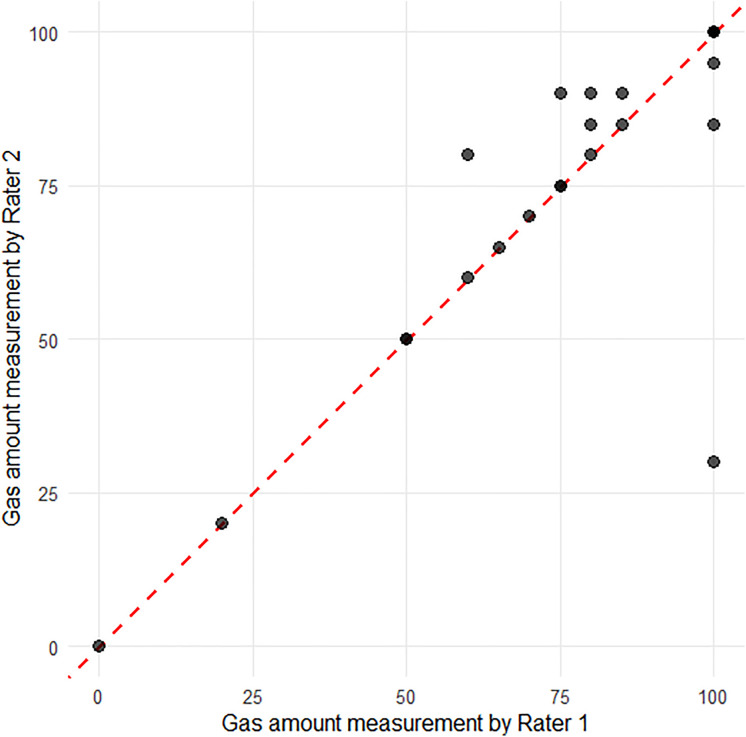
Interrater Agreement for the Amount of Air or Gas in the Anterior Chamber (AC). The graph shows the interrater agreement for measuring the amount of air or gas in the AC using the smart eye camera. Intraclass correlation coefficient = 0.817 [95% confidence interval: 0.616, 0.918]. There was little difference between the two raters. The dashed red line represents perfect agreement (*y* = *x*).

The judgments of the two raters regarding the amount of air or gas in the AC were consistent using the SEC (ICC = 0.817 [95% confidence interval (CI): 0.616, 0.918]) (Fig 2).

### Presence of pupillary block and graft detachment

Interrater agreement for pupillary block was good (Table 2), although a difference in the assessment of pupillary block was observed between the two raters with the SEC (Kappa coefficient = 0.646). In contrast, it was difficult to assess minor graft detachment using the SEC. Neither examiner could identify graft detachment using the SEC (Table 3).

**Table 2:** Interrater Agreement: Pupillary Block.■.

Method = Slit-lamp									
			Rater 2					Measure of agreement	
			[0] No		[1] Yes		Total	Point estimate [95% CI]	
**Rater 1**	[0] No		22 (95.7%)		0 (0%)		22 (95.7%)	% agreement	100% [85.2%, 100%]
	[1] Yes		0 (0%)		1 (4.3%)		1 (4.3%)	Kappa coefficient	1.000 [1.000, 1.000]
	Total		22 (95.7%)		1 (4.3%)		23 (100.0%)		
Method = Smart eye camera									
				**Rater 2**				**Measure of agreement**	
				[0] No		[1] Yes	Total	Point estimate [95% CI]	
**Rater 1**		[0] No		21 (91.3%)		0 (0%)	21 (91.3%)	% agreement	95.7% [78.1%, 99.9%]
		[1] Yes		1 (4.3%)		1 (4.3%)	2 (8.7%)	Kappa coefficient	0.646 [−0.032, 1.000]
		Total		22 (95.7%)		1 (4.3%)	23 (100.0%)		

Rater 1 = Expert ophthalmologist, Rater 2 = Early career ophthalmologist. CI, confidence interval.

**Table 3:** Interrater Agreement: Graft Detachment.

Method = Slit-lamp												
				Rater 2						Measure of agreement		
				[0] No		[1] Yes		Total		Point estimate [95% CI]		
**Rater 1**		[0] No		22 (95.7%)		0 (0%)		22 (95.7%)		% agreement		100% [85.2%, 100%]
		[1] Yes		0 (0%)		1 (4.3%)		1 (4.3%)		Kappa coefficient		1.000 [1.000, 1.000]
		Total		22 (95.7%)		1 (4.3%)		23 (100.0%)				
Method = Smart eye camera												
			**Rater 2**						**Measure of agreement**			
			[0] No		[1] Yes		Total		Point estimate [95% CI]			
**Rater 1**	[0] No		23 (100%)		0 (0%)		23 (100%)		% agreement		100% [85.2%, 100%]	
	[1] Yes		0 (0%)		0 (0%)		0 (0%)		Kappa coefficient		Not applicable	
	Total		23 (100%)		0 (0%)		23 (100.0%)					

Rater 1 = Expert ophthalmologist, Rater 2 = Early career ophthalmologist. CI, confidence interval.

**Fig 3:**
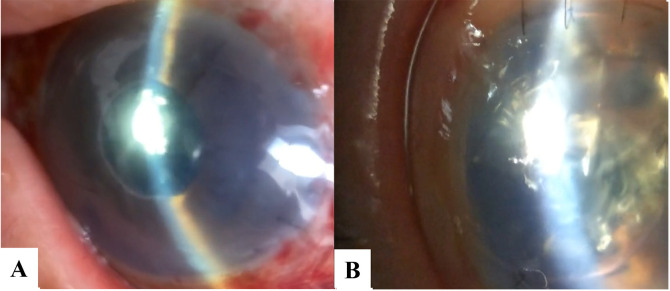
Evaluation of pupillary block and graft detachment using the SEC. (A) Postoperative evaluation using the SEC clearly shows a shallow anterior chamber due to a dislocation of tamponade gas behind the iris, indicating pupillary block. (B) Conversely, assessing graft detachment is hindered when the area of detachment is restricted. The patient was examined in a supine position at the bedside from the examiner’s temporal position.

No difference in identifying the presence of pupillary block or graft detachment was found between the two raters using the conventional slit-lamp. The images of pupillary block and graft detachment obtained with the SEC are shown in Fig 3.

Table 4 shows the sensitivity and specificity of the SEC for determining the presence of pupillary block and graft detachment. Conventional slit-lamp examination was used as the reference standard. The SEC demonstrated high sensitivity (100% [95% CI: 2.5, 100%]) and specificity (95.5% [95% CI: 77.2, 99.9%]) for detecting pupillary block. For graft detachment, it showed 100% specificity [95% CI: 84.6, 100%], but 0% sensitivity [95% CI: 0–97.5%] (Table 5).

**Table 4:** Performance of the Smart Eye Camera for Identifying the Presence of Pupillary Block.

		Smart eye camera			Performance	
Rater	Slit-lamp	[0] No	[1] Yes	Total	Point estimate [95% CI]	
1	[0] No	21 (95.5%)	1 (4.5%)	22 (100.0%)	Specificity	95.5% [77.2%, 99.9%]
	[1] Yes	0 (0%)	1 (100%)	1 (100.0%)	Sensitivity	100% [2.5%, 100%]
2	[0] No	22 (100%)	0 (0%)	22 (100.0%)	Specificity	100% [84.6%, 100%]
	[1] Yes	0 (0%)	1 (100%)	1 (100.0%)	Sensitivity	100% [2.5%, 100%]

Slit-lamp examination was used as the reference standard.

**Table 5:** Performance of the Smart Eye Camera for Identifying the Presence of Graft Detachment.

		Smart eye camera			Performance	
Rater	Slit-lamp	[0] No	[1] Yes	Total	Point estimate [95% CI]	
1	[0] No	22 (100%)	0 (0%)	22 (100.0%)	Specificity	100% [84.6%, 100%]
	[1] Yes	1 (100%)	0 (0%)	1 (100.0%)	Sensitivity	0% [0%, 97.5%]
2	[0] No	22 (100%)	0 (0%)	22 (100.0%)	Specificity	100% [84.6%, 100%]
	[1] Yes	1 (100%)	0 (0%)	1 (100.0%)	Sensitivity	0% [0%, 97.5%]

Slit-lamp examination was used as the reference standard.

## Discussion

In the present study, we were able to accurately assess graft detachment and pupillary block in most patients post-keratoplasty using SEC evaluations compared with using slit-lamp examinations. Regarding the safety and effectiveness of the smartphone-attachable SEC device, it functioned adequately for evaluating patients immediately after corneal transplantation on the day of surgery.

An important feature of the SEC is that images obtained post-surgery can be shared with colleagues through a platform that permits examiners to view all images simultaneously from any location. As a result, clinicians can quickly and efficiently exchange opinions regarding the need for additional treatments, such as observation, air injection, or secondary surgery. For example, if graft detachment is observed, the next procedure, including rebubbling or regrafting, must be considered.

Herein, three postoperative status factors were evaluated following lamellar keratoplasty. First, the amount of air or gas in the AC was judged by two raters. The amount of air or gas in the AC is essential. If too much air or gas is present (about 100%), the amount of air or gas should be reduced to avoid pupillary block. In contrast, no air or gas indicates a high risk of detachment, because no support is provided by underlying air.

Second, the presence of pupillary block was determined by the two raters. Pupillary block is a sight-threatening complication that can occur on day one postoperatively and must be resolved by prompt removal of air or gas from the AC. In the current study, despite a lack of evaluation precision using images with high resolution, we were able to judge the need for an anterior chamber air removal in emergency cases, such as high pressure due to pupillary block. The discrepancy between the two examiners was due to overestimations, resulting in further evaluation. Nonetheless, this discrepancy did not affect sight-threatening complications.

Third, the two raters identified the presence of graft detachment. Despite the successful outcomes for evaluating the amount of air or gas in the AC and the presence of pupillary block mentioned above, assessing the need for rebubbling to treat graft detachments with the SEC was challenging due to the presence of underlying air supporting the grafts. Hence, this may be a limitation of the SEC device. As shown in previous reports, AS-OCT is required for precise evaluation of minor graft detachment [5,6]. Given the lack of precise graft detachment evaluations with the SEC, AS-OCT and slit-lamp examinations should be performed on the following day to assess the need for rebubbling. Regardless, the use of the smartphone-attachable SEC enabled us to detect emergencies, such as pupillary block or a free-floating graft due to the absence of air or gas in the AC. Especially, pupillary block is a sight-threatening complication that can cause blindness [15]. Accordingly, utilizing a smartphone-attachable camera may be practical and beneficial for performing accurate evaluations post-surgery with less effort at night.

Our study has some limitations. First, as this was a pilot study, only a small number of patients were included. Another limitation was the inferior resolution quality of SEC images compared with AS-OCT images. Larger prospective studies are required to validate the use of the SEC device in patients undergoing keratoplasty and for examining patients at community hospitals, emergency or after-hours care, or in countries with limited resources [9,16].

In conclusion, the smartphone-attachable SEC was generally effective in the postoperative evaluation of patients who underwent keratoplasty. In particular, it was useful for assessing the success of surgery or identifying complications that required additional procedures.

## Supporting information

S1 Video Checking the eyes of a patient on day 0 after lamellar corneal transplantation. This video demonstrates the condition of the patient’s eye following lamellar keratoplasty. The amount of air in the anterior chamber, depth of the anterior chamber, and presence of pupillary block are clearly visible.
(MP4)
